# Lipidome Unsaturation Affects the Morphology and Proteome of the *Drosophila* Eye

**DOI:** 10.1021/acs.jproteome.3c00570

**Published:** 2024-03-14

**Authors:** Mukesh Kumar, Canan Has, Khanh Lam-Kamath, Sophie Ayciriex, Deepshe Dewett, Mhamed Bashir, Clara Poupault, Kai Schuhmann, Henrik Thomas, Oskar Knittelfelder, Bharath Kumar Raghuraman, Robert Ahrends, Jens Rister, Andrej Shevchenko

**Affiliations:** † Max Planck Institute of Molecular Cell Biology and Genetics, Pfotenhauerstrasse 108, Dresden 01307, Germany; ‡ Department of Biology, University of Massachusetts Boston, Integrated Sciences Complex, 100 Morrissey Boulevard, Boston, Massachusetts 02125, United States; § Department of Analytical Chemistry, University of Vienna, Vienna 1090, Austria

**Keywords:** Drosophila, retina, diet, membrane lipid unsaturation, proteome, phototransduction

## Abstract

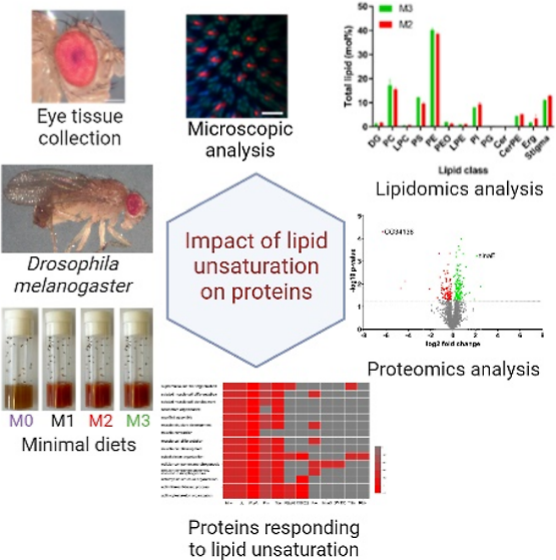

Organisms respond to dietary and environmental challenges by altering the molecular composition of their glycerolipids and glycerophospholipids (GPLs), which may favorably adjust the physicochemical properties of lipid membranes. However, how lipidome changes affect the membrane proteome and, eventually, the physiology of specific organs is an open question. We addressed this issue in *Drosophila melanogaster*, which is not able to synthesize sterols and polyunsaturated fatty acids but can acquire them from food. We developed a series of semisynthetic foods to manipulate the length and unsaturation of fatty acid moieties in GPLs and singled out proteins whose abundance is specifically affected by membrane lipid unsaturation in the *Drosophila* eye. Unexpectedly, we identified a group of proteins that have muscle-related functions and increased their abundances under unsaturated eye lipidome conditions. In contrast, the abundance of two stress response proteins, Turandot A and Smg5, is decreased by lipid unsaturation. Our findings could guide the genetic dissection of homeostatic mechanisms that maintain visual function when the eye is exposed to environmental and dietary challenges.

## 1 Introduction

The composition of an organism’s proteome is determined by its genome. In contrast, its lipidome and metabolome result from a complex and poorly understood interplay among diet, temperature, development, and metabolism. In free-living organisms, diet and temperature affect the length and unsaturation of fatty acid moieties in glycerophospholipids (GPLs)^1,2^ and control key membrane properties such as fluidity or lateral organization. However, it remains unclear if altered lipidome composition also leads to marked changes in the proteome, particularly affecting the abundance of lipid-binding or membrane-associated proteins. Here, we analyze the ocular proteome of *Drosophila melanogaster* as a model to gain insights into this potential lipidome–proteome interplay.

The *D. melanogaster* compound eye is a membrane-rich organ that contains relatively low levels of storage lipids such as di- and triacylglycerols (DAGs and TAGs, respectively).^3^ Flies only synthesize saturated or monounsaturated fatty acids because they lack Δ-6 and Δ-5 desaturases.^4^ However, others and we^1,3,5^ have previously noticed that fly eyes comprise a sizable proportion of dietary polyunsaturated fatty acids (PUFA), which is critical for vision.^6,7^ For instance, PUFA deficiency reduces the sensitivity of the photoreceptors, slows their photomechanical responses to visual stimuli,^6^ and impairs their synaptic transmission.^7^

Since the lipid composition and nutritional value of commonly used “standard” laboratory foods can differ substantially, we previously developed a yeast extract-based and low lipid content “M1” food medium (Figure S1) that we supplemented with the plant sterol stigmasterol and the vitamin A precursor beta-carotene.^2,8,9^ We showed that the morphology of the *Drosophila* compound eye and the light-sensing compartments of its photoreceptors (rhabdomeres) appeared wild type in flies reared on M1-food.^8^ Moreover, we recently studied the impact of vitamin A deficiency on the lipidome, proteome, and structure of the eye by omitting beta-carotene from M1-food (resulting in “M0-food”).^8^ Vitamin A deprivation damaged the rhabdomere morphology and decreased the abundance of phototransduction proteins. Remarkably, it had no effect on the eye lipidome.

Here, we manipulated the eye lipidome by using M1-food as the basis to generate two compositionally related food media that we supplemented with either an equal amount of synthetic saturated (“M3-food”) or polyunsaturated (“M2-food”) TAGs, respectively (Table 1). The M2- and M3-foods allowed us to raise flies whose eyes had a highly contrasting length and unsaturation of fatty acid moieties of major GPLs, while the molar abundance of other membrane lipids (e.g., sphingolipids or sterols) remained largely unchanged. By comparing the ocular proteome dynamics triggered by rearing *Drosophila* on four different, yet compositionally related, diets (M0–M3 foods), we identified groups of proteins whose abundances were specifically affected by lipid unsaturation.

**Table 1:** Overview of “M”-Food Composition^a^

food	yeast extract	stigmasterol	beta-carotene	TAG 42:0	TAG 66:18
				TAG 48:0	
				TAG 54:0	
M0^b^^,^^c^	x	x			
M1	x	x	x		
M2^b^	x	x	x		x
M3	x	x	x	x	

a Added component is designated with “x”.

b Flies reared on these foods showed decreased rhabdomere size.

c Reported in ref (8).

## 2 Experimental Section

### 2.1 Annotation of Lipid Species

Lipid classes were annotated^10^ as follows: Cer, ceramides; Cer-PE, phosphorylethanolamine ceramides; DAG, diacylglycerols; TAG, triacylglycerols; PE, phosphatidylethanolamines; PE*-O*, 1-alkyl,2-acylglycerophosphoethanolamine; PC, phosphatidylcholines; PG, phosphatidylglycerols; PI, phosphatidylinositols; and PS, phosphatidylserines. Species of GPL, DAG, and TAG lipid classes were annotated as lipid class ⟨no. of carbon atoms in all fatty acids⟩: ⟨no. of double bonds in all fatty acid moieties⟩. Sphingolipid species were annotated as lipid class ⟨no. of carbon atoms in the long-chain base and fatty acid moieties⟩: ⟨no. of double bonds in the long-chain base and fatty acid moieties⟩; ⟨no. of hydroxyl groups in the long-chain base and fatty acid moiety⟩.

### 2.2 *Drosophila* Culture

The *D. melanogaster* wild-type strains Oregon R and Canton S were reared under a 12 h light/12 h dark cycle at 25 °C. The flies were raised on one of the five different food media: standard food (SF) diet, see below, or M0, M1, M2, or M3 diets from the embryonic to the adult stage. 3-to-4 days old male flies were collected under brief anesthesia on a carbon dioxide pad and used for the experiments.

### 2.3 *Drosophila* Food Media

SF contained per liter 8 g of agar, 18 g of brewer’s yeast, 10 g of soybean, 22 g of molasses, 80 g of cornmeal, 80 g of malt, 6.3 mL of propionic acid (Sigma-Aldrich), and 1.5 g of Nipagin (Sigma-Aldrich). Minimal M0 food contained per liter 10 g of UltraPure Agarose (Invitrogen), 100 g of yeast extract (Kerry), 100 g of glucose (Merck), 1.5 mL of Nipagin (Sigma-Aldrich) in 10% in ethanol, and 1 g stigmasterol (Sigma). M1 food is M0 food supplemented with 0.5 g of β-carotene (Sigma). M2 food is M1 food supplemented with an unsaturated TAG (TAG 66:18; Larodan), and M3 is M1 food supplemented with equal amounts (27, 29, and 28 mg) of three saturated TAGs (TAG 42:0; TAG 48:0; TAG 54:0; all from Larodan, Sweden).

### 2.4 Imaging the *Drosophila* Eye

Adult eyes were imaged with a Stemi 508 Trinoc microscope (Zeiss model #4350649030) and an Axiocam 208 HD/4k color camera (Zeiss model #4265709000, set to auto exposure and auto white balance), as previously described.^8^ Briefly, flies were anesthetized with CO_2_ and transferred to a 60 mm Petri dish (Falcon) filled with about 10 mL of a liquid agarose gel, which was prepared by dissolving 2 g of ultrapure agarose (Invitrogen) in 100 mL of distilled water and heating to 58 °C. Images were processed with Fiji, Adobe Photoshop 2020, and Adobe Illustrator 2020 software.

### 2.5 Confocal Microscopy and Immunohistochemistry of *Drosophila* Photoreceptors

We visualized the photoreceptors of 4 day old flies, as previously described.^11^ Briefly, we dissected the adult eyes and fixed them in 3.8% formaldehyde solution before removing the lamina and head cuticle. The eyes were incubated overnight in mouse anti-Rh1 primary antibody (4C5, from Developmental Studies Hybridoma Bank, University of Iowa) that was diluted 1:10 in PBST (PBS + 0.3% Triton-X, Sigma). The next morning, the eyes were washed three times with PBST. In the evening, the eyes were incubated in secondary Alexa Fluor 647-conjugated antibody (raised in donkey, Invitrogen) diluted 1:800 in PBST and Alexa Fluor 488-conjugated Phalloidin (1:100, Invitrogen). The next morning, the eyes were washed three times with PBST. Lastly, the eyes were mounted with SlowFade (Molecular Probes) on a bridge slide and imaged with a Zeiss LSM 8 confocal microscope. Raw images were processed with Fiji (https://imagej.net/software/fiji/), Adobe Photoshop, and Adobe Illustrator.

### 2.6 Rhabdomere Measurements and Statistics

The cross-sectional area of the rhabdomeres was quantified using Phalloidin staining as previously described.^8^ Briefly, we used Fiji’s (https://imagej.net/software/fiji/) freehand selection tool to draw a circle around the cross-section of an R3 photoreceptor rhabdomere at the level of the R8 rhabdomeres. Next, we used the ROI manager tool to measure the area of the circled rhabdomere. For each food type, eight unit eyes from five different retinas were analyzed. We used RStudio (https://www.rstudio.com/) to perform an ANOVA, followed by Tukey’s Honestly Significance Difference Test (HSD) for pairwise comparisons, to determine whether the rhabdomeric size differences were statistically significant between different food media. The significance cutoff was *p* < 0.05; box-and-whisker plots were generated in RStudio.

### 2.7 Lipid Extraction and Shotgun Lipidomics Analysis of the *Drosophila* Eye

Whole eyes (*n* = 10) were dissected with a thin blade and placed in 40 μL of 150 mM ammonium bicarbonate buffer containing 10% of isopropanol (IPA) into a 2 mL Eppendorf tube, snap-frozen in liquid nitrogen, and stored at −80 °C or processed immediately. The eyes were mechanically disrupted with 1 mm zirconia beads, and samples were dried under vacuum to remove isopropanol. The total lipids were extracted using the methyl *tert*-butyl ether (MTBE) extraction according to ref (12). Samples were resuspended in 200 μL of water and 700 μL of MTBE/methanol (5:1.5, v/v) containing internal standards (0.539 nmol zymosterol-d5, 0.782 nmol stigmasterol-d6, 0.313 nmol triacylglycerol-d5 50:0, 0.073 nmol diacylglycerol-d5 34:0, 0.138 nmol PC 12:0/13:0, 0.109 nmol LPC 13:0, 0.067 nmol PS 12:0/13:0, 0.147 nmol PE 12:0/13:0, 0.053 nmol LPE 13:0, 0.090 nmol PI 12:0/13:0, 0.068 nmol PG 12:0/13:0, 0.102 nmol Cer 30:1, 0.077 nmol PA 12:0/13:0, 0.068 nmol GalCer 30:1, 0.081 nmol LacCer 30:1, and 0.074 nmol CerPE 29:1). After centrifugation, the organic phase was collected and dried under a vacuum to avoid lipid oxidation. The whole extraction procedure, including sample preparation, was performed at 4 °C in order to prevent lipid degradation. Mass spectrometric analyses were performed on a Q Exactive instrument (Thermo Fisher Scientific, Bremen) equipped with a robotic nanoflow ion source TriVersa NanoMate (Advion BioSciences, Ithaca, NY) using chips with spraying nozzles with a diameter of 4.1 μm. Lipids were identified by LipidXplorer software^13^ by matching *m*/*z* of their monoisotopic peaks to the corresponding elemental composition constraints.

### 2.8 Protein Extraction and GeLC-MS/MS Analysis of the *Drosophila* Eye Proteome

The compound eyes (*n* = 40) were dissected from the male flies raised under different food conditions (see above) and placed in lysis buffer containing 150 nM NaCl, 1 mM EDTA, 50 mM Tris–HCl (pH 7.5), 1 tablet Roche protease inhibitors, 0.2% w/v CHAPS, 0.1% w/v OGP, 0.7% v/v triton X-100, and 0.25 μg/mL DNase and RNase. The samples were immediately snap frozen using liquid nitrogen, stored at −80 °C or immediately processed. The eye tissues were homogenized, and to the supernatant an equal volume of 2× SDS Laemmli sample buffer (SERVA Electrophoresis GmbH, Heidelberg, Germany) was added. The samples were heated at 80 °C for 10–15 min and then loaded on 4–20% 1D SDS PAGE (Anamed Elektrophorese, Rodau, Germany). The protein bands were visualized by Coomassie Brilliant Blue staining. Each gel lane was cut into six gel slices, and each gel slice was codigested with heavy isotope labeled CP02 and a gel band containing 1 pmol bovine serum albumin (BSA) standard.

### 2.9 GeLC-MS/MS

In-gel digestion was carried out as previously described.^8^ Briefly, the electrophoresed gel rinsed with water was stained with Coomassie Brilliant Blue R-250 for 10 min at RT and then destained with destaining solution (water/methanol/acetic acid, 50:40:10 (v/v/v). The gel slice was excised according to the expected molecular weight of the proteins of interest and further cut into small pieces (∼1 mm size). The gel pieces were then transferred into 1.5 mL LoBind Eppendorf tubes and further processed. The gel pieces were completely destained by ACN/water, and reduction was done by incubating the gel pieces with 10 mM dithiothreitol at 56 °C for 45 min. Alkylation was carried out with 55 mM iodoacetamide for 30 min in the dark at RT. The reduced and alkylated gel pieces were washed with water/ACN and finally shrunken with ACN; ice-cold trypsin (10 ng/μL) was added to cover the shrunken gel pieces, and after 1 h of incubation on ice, excess trypsin (if any) was discarded. The gel pieces were then covered with 10 mM NH_4_HCO_3_ and incubated for 12–15 h at 37 °C. The tryptic peptides were extracted using water/ACN/FA, dried using a vacuum centrifuge, and stored at −20 °C until next use. The tryptic peptides were recovered in 5% aqueous FA, and 5 μL were injected using an autosampler into a Dionex Ultimate 3000 nano-HPLC system, equipped with a 300 μm i.d. × 5 mm trap column and a 75 μm × 15 cm Acclaim PepMap100 C18 separation column. 0.1% FA in water and ACN were used as solvents A and B, respectively. The samples were loaded on the trap column for 5 min with a solvent A flow of 20 μL/min. The trap column was then switched online to the separation column, and the flow rate was set to 200 nL/min. The peptides were fractionated using a 180 min elution program: a linear gradient of 0 to 30% B delivered in 145 min, and then B % was increased to 100% within 10 min and maintained for another 5 min, dropped to 0% in 10 min, and maintained for another 10 min. Mass spectra were acquired using a Q Exactive HF mass spectrometer (Thermo Fisher Scientific, Bremen, Germany).

### 2.10 Absolute Quantification of Proteins by MS Western

MS Western (MSW) for the quantification of target protein was carried out as previously described.^8,14^ Briefly, the gel slice from each sample was codigested with a heavy isotope-labeled MSW standard and a gel band containing a known amount (1 pmol) of BSA protein. Molar abundance of 43 target proteins (Table S3) was inferred from the corresponding heavy-labeled peptides (multiple peptides for each target protein were used^15^ from the MSW standard). The quantity of the MSW standard was in turn referenced to the known molar amount of the BSA standard.

### 2.11 Data Processing for Protein Identification and Quantification

Mascot v2.2.04 (Matrix Science, London, UK) was used for peptide identifications against the custom-made database containing the sequence of the target protein, to which sequences of human keratins and porcine trypsin were added. For eye proteome analysis, the *Drosophila* reference proteome database from UniProt^16^ was used. The database searches were performed with the following mascot settings: precursor mass tolerance of 5 ppm; fragment mass tolerance of 0.03 Da; fixed modification: carbamidomethyl (C); variable modifications: acetyl (protein N-terminus) and oxidation (M); label: 13C (6) (K), label: 13C (6) 15N (4) (R), 2 missed cleavages were allowed. Progenesis LC–MS v4.1 (nonlinear dynamics, UK) was used for the peptide feature extraction, and the raw abundance of identified peptide was used for absolute quantification. MaxQuant version 1.5.5.1 and Perseus version 1.5.5.3 were used for label-free quantification and subsequent statistical analysis. MaxQuant analysis was performed with default settings. Gene ontology (GO) term analysis was performed by mapping the regulated genes to ontology terms to identify the over-represented categories.^17^

The mass spectrometry proteomics data have been deposited to the ProteomeXchange Consortium via the PRIDE partner repository^18^ with the data set identifier PXD044999.

## 3 Results

### 3.1 Experimental Rationale and Design of Synthetic Food Media for Dietary Interventions

We aimed to delineate how manipulations of lipid unsaturation affect the *D. melanogaster* eye proteome. To this end, we altered the unsaturation of the eye lipidome by rearing flies on a set of four compositionally related food media (M0, M1, M2, and M3; Table 1).

As their basis, we used M1-food, a soluble budding yeast extract with low lipid content that we supplemented with the vitamin A precursor beta-carotene^9^ and the phytosterol stigmasterol^8^ (Figure S1). M1-food thus provided an optimal starting composition for targeted dietary interventions. To supply flies with saturated and unsaturated dietary fatty acids, we supplemented M1-food with an equal amount of unsaturated (M2-food; TAG 22:6/22:6/22:6) or saturated TAGs (M3-food; mixture of synthetic TAG 14:0/14:0/14:0, TAG 16:0/16:0/16:0, and TAG 18:0/18:0/18:0 taken in an equal (w/w) proportion) (Table 1). Effectively, M2-food supplied docosahexaenoic acid (DHA), an omega-3 fatty acid that flies can metabolize to C20:5, C20:4, and even shorter PUFAs, and use them for the synthesis of GPLs.^4^ Conversely, M3-food resembled a “common fly diet”^3^ that supplies the flies with medium-length saturated fatty acids such as C14:0, C16:0, and C18:0. Consistent with this dietary similarity, medium-chain saturated TAGs are common in *D. melanogaster*.^3,19^ In contrast, M2-food supplied unsaturated fatty acids that flies cannot generate de novo but that can be metabolically derived from C22:6.^4^ In both M2- and M3- foods, the relative (w/w) content of the TAGs was the same. For clarity, we will hereafter refer to flies that were reared on the respective “M-” food as “M-” flies. Our goal was not to correlate lipidome and proteome responses to the exact composition of the M-foods; instead, we used M-foods as a tool to achieve the desired eye lipidome composition that we validated by shotgun mass spectrometry analyses.

### 3.2 Lipidome and Proteome of M3-Flies

**Figure 1:**
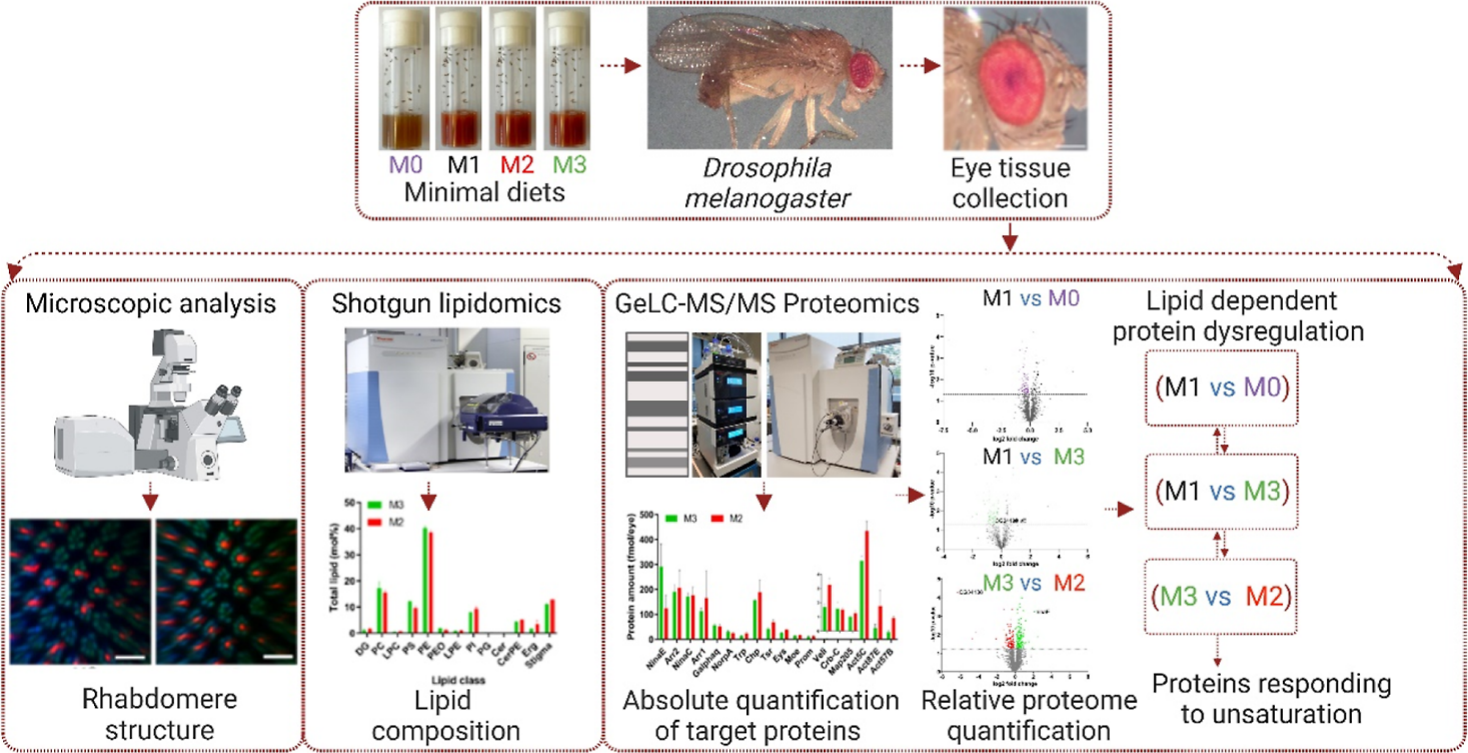
Experimental workflow to determine how the unsaturation of membrane lipids affects the ocular morphology and proteome in flies. Eyes were dissected from flies reared on the four M-foods (Table 1); the rhabdomere structure was analyzed by confocal microscopy; lipid and protein compositions were quantified by mass spectrometry. Targeted absolute quantification reported molar abundance (per eye) of key proteins involved in phototransduction and rhabdomere maintenance; relative (fold change) quantification revealed global changes between ocular proteomes of different M-flies. Comparison of trends of proteome changes in different M-flies revealed proteins specifically responding to unsaturation but not to other factors (e.g., TAG content or altered morphology).

**Figure 2:**
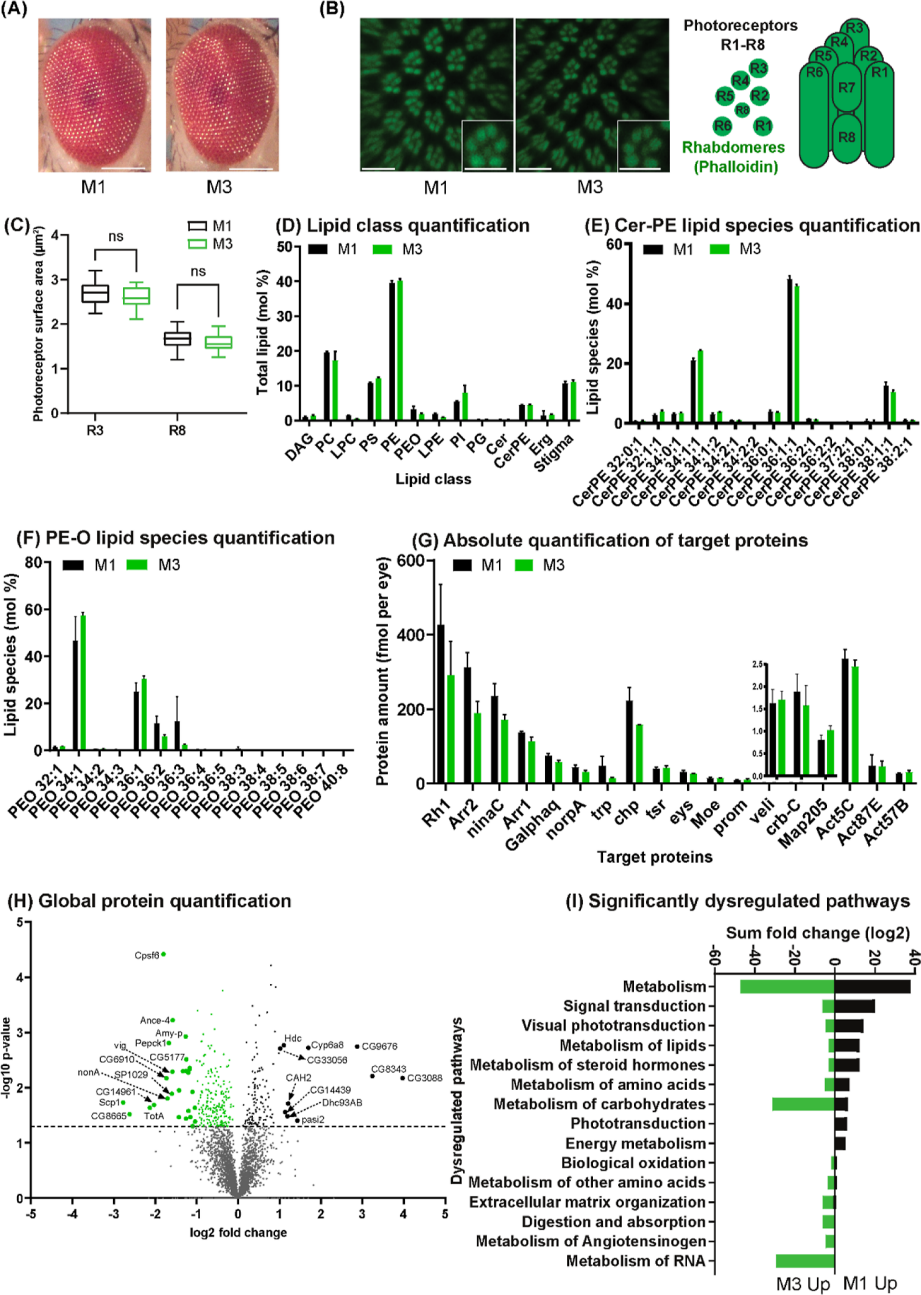
Ocular morphology, proteome, and lipidome of M3- and M1-flies. (A) Normal external compound eye morphology in M3- and M1-flies. Scale bars: 70 μm. (B) Confocal whole-mount images of male retinas show seven rhabdomeres (phalloidin, green) in each unit eye. Insets show single unit eyes. Scale bars: 10 μm. Schematic shows the cross-section (left) and side view (right) of the rhabdomeres of the photoreceptors R1–R8 of a single unit eye (ommatidium). (C) Cross-sectional areas (μm^2^) of the rhabdomeres of two different photoreceptor types (outer: R3, inner: R8) are not statistically different (unpaired *t* test, p value < 0.1690 and <0.3592 for R3 and R8, respectively) in M3- and M1-flies. (D) Lipid class composition (mol %) of the eye. (E) Profile of Cer-PE molecular species. (F) Profile of PE*-O* molecular species. (G) MSW quantification of the absolute (molar) abundances of photoreceptor morphology or phototransduction proteins reveals significant differences. (H) Volcano plot showing differentially expressed ocular proteins between M3- and M1-flies. (I) GO term enrichment analysis of significantly (*p*-value ≤ 0.05) dysregulated pathways. The error bars in graphs in Panels (D–G) represent mean with SD.

We previously reported that the morphology of the compound eyes and the light-sensing compartments of the photoreceptors (rhabdomeres) (Figure 2A,B)^20^ of flies raised on M1-food closely resembled flies raised on “standard” lab food (SF, Experimental Section).^8^ Next, we used the M1-derived M2- and M3-foods (Table 1) to generate and compare the morphology, lipidome, and proteome composition of the eyes of flies with drastically different lipid unsaturation (Figure 1).

First, we tested whether M3-food (low-lipid content M1-food supplemented with saturated TAGs) affected the eye morphology, lipidome, and proteome (Figure 2). The M3 diet caused no obvious structural eye defects: the external compound eye morphology (Figure 2A) as well as the rhabdomere structure and arrangement (Figure 2B) were very similar to those of M1- and SF-flies.^8^ Moreover, the rhabdomere cross-sectional area of the outer and inner photoreceptor types^21^ was also not affected (*p* > 0.05; Figure 2C).

**Figure 3:**
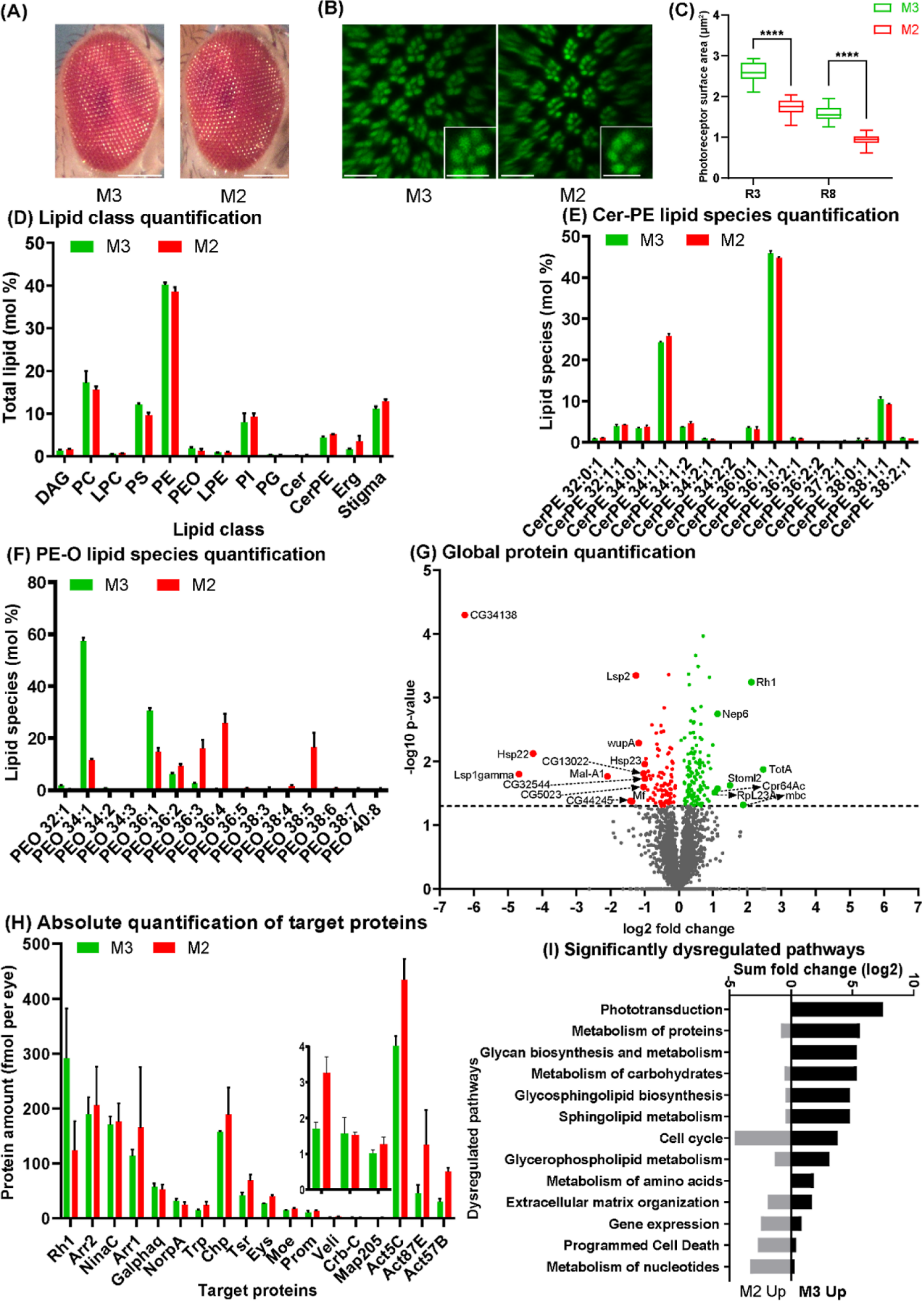
Ocular morphology, proteome, and lipidome of M2- and M3-flies. (A) Normal external compound eye morphology for both dietary conditions. Scale bars, 70 μm. (B) Confocal whole-mount images of male retinas show seven rhabdomeres (Phalloidin, green) that are thinner in M2-flies. Insets show single unit eyes; note that the M2-fly rhabdomeres are thinner. Scale bars: 10 μm. (C) Quantification of the cross-sectional areas (μm^2^) of the rhabdomeres of two different photoreceptor types (outer: R3, inner: R8) reveals significantly (unpaired *t* test, *p* value < 0.0001 and <0.0001 for R3 and R8, respectively) reduced rhabdomeres in M2-flies. (D) Lipid class composition (mol %) of the eye is similar between M2- and M3-flies. (E) Profile of Cer-PE molecular species is similar between M2- and M3-flies. (F) Profile of PE*-O* molecular species is significantly different. (G) Volcano plot showing differentially expressed proteins. The CG34138 protein is highly upregulated in M2-flies. (H) MSW quantification of the absolute (molar) abundances of photoreceptor morphology or phototransduction proteins. (I) GO term enrichment analysis of significantly (*p*-value ≤ 0.05) dysregulated pathways. The error bars in graphs in Panels Figure 2D–H represent mean value with SD.

Next, we subjected eyes collected from ten M1- and M3-flies, respectively, to shotgun lipidomics and quantified the molar abundance of 243 lipid species from 11 lipid classes, including the major membrane lipid classes LPE, PE, PE-*O*, Cer, Cer-PE, PC, LPC, PS, PI, DAG, and PG (Table S1). We found no significant difference in the mol % of lipid classes (Figure 2D) or the lipid species composition of sphingolipids (Figure 2E) and GPLs (Figure 2F). For clarity, we only present molecular species profiles of Cer-PE and of the brain-specific class of GPL PE*-O* (Figures 2 and 3);^3^ other GPLs classes were also unaffected by M1-food. The full lipidome composition (all quantified species from all lipid classes) of all M-flies is available in Table S1. Taken together, supplementation of M1-food with saturated TAGs had no apparent impact on the composition and content of major lipid classes in the eye.

Full proteome analysis of the eyes of M3-flies by GeLC-MS/MS revealed that the abundances of 221 proteins were increased and those of 120 proteins decreased (Figure 2H) as compared to M1-flies (Table S2). Subsequent pathway analysis indicated that the affected proteins (respective numbers are in parentheses) were assigned to “metabolism of proteins” (15), “metabolism of carbohydrates” (16), “metabolism of lipids” (5), “metabolism of RNA” (11), “signal transduction” (11), and “phototransduction” (6). We interpret these data such that the added TAGs led to a higher caloric value of M3-food and thus to an expected metabolic response (Figure 2I).

Since we observed no apparent changes in photoreceptor morphology or Rhodopsin expression, we next analyzed whether the saturated TAGs perturbed the molar ratios between specific photoreceptor proteins. We employed our MSW method^14^ to determine the absolute (molar) abundances of 43 proteins that play key roles in phototransduction (Figure S2) as well as the development and maintenance of photoreceptors^8,22^ (Table S3). Notably, compared to M1-flies, the abundance of a few phototransduction proteins,^23^ like Rh1 (Rhodopsin 1), Arr2 (Arrestin 2), NinaC (neither inactivation nor afterpotential C), and Trp (transient receptor potential) decreased by 1.4- to 1.6-fold in M3-flies without significant changes in their relative molar ratios (Figure 2G and Table S3). In contrast, the abundances of other phototransduction proteins such as Arr1 (Arrestin 1), Galphaq (G protein alpha subunit q), NorpA (No receptor potential A), and morphology-related proteins such as Actins (Act5C, Act87E, Act57B), Veli, Prom (Prominin), Moe (Moesin), Eys (Eyes shut), and Crb-c (Crumbs) were unchanged (Figure 2G and Table S3), consistent with the wild-type morphology of the compound eyes and the photoreceptors (see above). Since both phototransduction and morphology-related proteins were quantified in the same experiment, this indicates that the same rate of decrease in the abundance of the former proteins was not because of normalization or sample processing errors.

We conclude that the dietary supply of TAGs with medium-chain saturated fatty acid moieties, which are compositionally similar to common TAGs in *Drosophila*,^3,19^ neither impacts the eye morphology nor the content of major lipid classes in the eye. However, these supplemented TAGs affected several metabolic pathways and decreased the molar abundance of major phototransduction proteins.

### 3.3 M2-Flies Show a Perturbed Photoreceptor Morphology

To analyze the impact of increased unsaturation of the ocular lipidome, we reared flies on M2-food that contained a synthetic TAG with three moieties of docosahexaenoic acid (DHA, C22:6). While M2-flies had a normal external eye morphology (Figure 3A), their rhabdomere cross-sectional area was significantly reduced compared to M3-flies (*p* < 0.0001; Figure 3B,C) and M1-flies (see above).^8^ Taken together, oversupplying a polyunsaturated TAG decreased the rhabdomere size in M2-flies.

### 3.4 Eye Lipidome of M2-Flies is Highly Unsaturated

Despite the major difference in unsaturation between the supplemented dietary TAGs, the ocular lipid class composition of M1-,^8^ M2-, and M3-flies was surprisingly similar (Figure 3D). However, the molecular species profile in M2-flies changed in a lipid class-dependent manner: GPLs incorporated a variety of PUFAs that were metabolically derived from the DHA moieties of the supplied unsaturated TAG. We detected polyunsaturated species with up to six double bonds in both fatty acid moieties in all major GPL classes, PC, PE, PS, PI, and PG (Table S1), but also in brain-specific PE*-O*^3^ (Figure 3F). Furthermore, the absolute abundance of shorter and more saturated lipids (zero to two double bonds per lipid molecule) was significantly reduced. In contrast, the molecular profiles of Cer-PE (Figure 3E), *lyso-*PC, and *lyso-*PE, but also of major energy storage lipids such as DAG, were largely unaffected (Table S1). This may suggest that the metabolic conversion of DHA to shorter PUFAs could be a rate-limiting step in resynthesizing endogenous lipids. Lastly, supplying an unsaturated TAG did not increase the production of sphingolipids with elongated or unsaturated N-acylamidated fatty acid moieties. Altogether, the ocular membrane lipidome of M2-flies became markedly unsaturated because its GPL component incorporated diet-derived PUFAs.

### 3.5 Proteome-Wide Impact of Lipidome Unsaturation

To assess the proteome-wide impact of membrane lipidome unsaturation, we compared the ocular proteomes of M2- and M3-flies. Out of the total of 3106 quantified proteins, 99 were significantly upregulated and 127 downregulated in M2-flies. Remarkably, Rh1 was downregulated in M2-flies (Figure 3G,H), while CG34138 was strongly upregulated (Figure 3G). We previously observed the same trend in M0-flies that have perturbed rhabdomere morphology but the same lipidome as M1-flies.^8^ In both M2- vs M3- and M1- vs M3-flies (Figures 2H and 3G), the protein changes were mostly associated with altered metabolism and affected similar metabolic pathways (Figures 2I and 3I), albeit with a different fold-change magnitude. In contrast to M1- and M3-flies, both the lipidome and the rhabdomere morphology were affected in M2-flies (Figure 3B,C). Conversely, we previously showed that vitamin A deprivation by M0-food (i.e., M1-food lacking the vitamin A precursor beta-carotene; Table 1) also affected rhabdomere morphology but not the eye lipidome.^8^ We therefore reasoned that the ocular proteome composition is influenced by three main factors (summarized in Table 2): (i) rhabdomere morphology defects; (ii) global changes in metabolism due to the added TAGs, irrespective of their fatty acid moieties; and (iii) increased unsaturation of membrane lipids caused by incorporation of PUFAs derived from unsaturated dietary TAG.

**Table 2:** Rationale behind the Successive Subtraction of Proteomic Data Sets^a^

comparison of proteomes^b^	reflects impact of		
	supplied TAGs	decreased rhabdomere size	unsaturated GPLs
M3 vs M2	Y	Y	Y
M1 vs M0	N	Y	N
M3 vs M1	Y	N	N

a The impact of factors revealed by the pairwise comparison is marked with “Yes (Y) and No (N)”.

b Comparison of the proteomes of corresponding M-flies.

We further hypothesized that the proteome changes in M2-flies (as compared to M1-flies) overlap with those of M0-flies because both dietary manipulations decreased the rhabdomere size. Lastly, we expected that proteome changes in M2- and M3-flies when compared to those in M1-flies might overlap because of metabolic response to a lipid-rich diet. Hence, we took advantage of these four (M0-, M1-, M2-, and M3- flies) proteomic data sets to perform a pairwise comparison and successive subtraction of similarly regulated proteins to identify those whose abundance specifically responds to membrane lipid unsaturation.

### 3.6 Identification of Proteins that Respond to Lipidome Unsaturation

To identify ocular proteins that respond to lipidome unsaturation, we first filtered the data sets with two permissive cutoff thresholds for *p-*value (*p* < 0.05 or −log 10 > 1.3) and for fold change (|FC| > 1.5 or |log2 FC| > 0.58), where ± indicates the direction of change in the M-food comparison (Table S4). For clarity, we assigned a negative FC to proteins enriched in M*y*- flies in the M*x-*flies vs M*y-*flies comparison. Next, from the list of significantly changed proteins in the M3-flies vs M2-flies comparison (Table S5) (hereafter termed [M3 vs M2]), we subtracted the list [M1 vs M0], which yielded the {[M3 vs M2] – [M1 vs M0]} list. During subtraction, we chose to (i) disregard actual |FC| values (we underscore that all proteins met |FC| > 1.5 threshold) and (ii) only subtracted proteins whose abundance changed in the same direction in both comparisons. For example, a protein was removed from the [M3 vs M2] list if it was upregulated in M2-flies but also in M0-flies in the [M1 vs M0] comparison (Table S6), irrespective of the |FC| magnitude. Hence from the [M3 vs M2] list, we removed proteins whose abundance changed in the same direction by lipidome-independent abnormal rhabdomere morphology and thus could not be attributed to a specific response to lipidome unsaturation (Table 2). Notably, filtering removed some highly regulated and abundant proteins (e.g., Rh1 and CG34138) because they responded in the same way to lipidome-dependent [M3 vs M2] and lipidome-independent [M1 vs M0] manipulations. Next, we applied the same criteria to subtract proteins that similarly responded to adding saturated TAGs ([M3 vs M1]) (Table S2), i.e., they were not affected by TAG unsaturation. Altogether, the final list is {[M3 vs M2] – [M1 vs M0] – [M1 vs M3]} (Table S4), comprising 67 proteins that are likely to be affected by ocular lipidome unsaturation, and surprisingly, only 15 of these proteins were membrane-associated. In comparison, out of the 3106 proteins quantified in M2-flies, 910 proteins were membrane-associated.

Strikingly, the higher unsaturation of membrane lipids did not impact the abundance of membrane proteins *en masse*. Within this final list, we recognized protein groups that share common trends in the unsaturation response:

#### Proteins Whose Abundance Increased with Lipidome Unsaturation but Were Unresponsive to the Other Dietary Manipulations

A group of seven proteins was upregulated in M2-flies (unsaturated) but unchanged in M0-flies (vitamin A-deprived) and M3-flies (saturated TAG). Unexpectedly, a GO term analysis^17^ revealed that all these proteins have muscle-related functions (Figure S3). This group comprises Myofilin (a structural component of the thick muscular filaments);^24^ Paxillin (a cytoskeletal adaptor protein that regulates cell fusion in muscles);^25^ Paramyosin (an invertebrate-specific muscle protein that is part of the thick filament); Myosin heavy chain; Upheld (encodes the calcium-binding muscle regulatory protein Troponin T);^26^ Wings up A (a cytoskeletal protein of the Troponin complex); and RIM-binding protein (an active zone protein involved in neuromuscular synaptic transmission).

#### Proteins Whose Abundance Decreased with Lipidome Unsaturation but Increased by Saturated TAGs

Conversely, our analysis identified a group of six proteins whose abundance decreased in “unsaturated” M2-flies and increased in “saturated” M3-flies. This protein group includes the putative TAG lipase CG5162, whose human orthologues are implicated in hyperlipidemia and obesity, and two lipid transporter proteins; the scaffolding apolipoprotein apolipophorin is a member of the conserved ApoB family and is involved in the synthesis of the lipoprotein lipophorin, which transports lipids between tissues. Crossveinless d is another lipoprotein that resembles vitellogenins, a component of the embryonic yolk of insects and a lipoglycoprotein that is synthesized and stored in the fat body.^27^ Notably, this group also included two proteins that mediate stress responses, Turandot A (TotA) and Smg5. The humoral factor TotA exhibited the strongest (over 4-fold) downregulation among all proteins in response to lipidome unsaturation and has been shown to respond to various types of environmental stresses.^28^ Smg5 is an essential nonsense-mediated mRNA decay factor that was found in an obesity screen to regulate TAG levels specifically in muscle cells.^29,30^ Lastly, the two predicted plasma membrane metallopeptidases angiotensin-converting enzymes Ance-4 and Neprilysin 6 were both downregulated in M2-flies, albeit Neprilysin 6 was not significantly changed in M3-flies.^31,32^

#### Proteins Whose Abundance Increased with Lipidome Unsaturation but Decreased by Saturated TAGs

Interestingly, the 130 kDa-Golgi matrix protein GM130 showed the reverse lipid response pattern: it was downregulated in the eyes of M3-flies but upregulated in M2-flies. GM130 is a structural protein that is involved in connecting the Golgi compartments in the soma and dendrites of neurons.^33^

Finally, we identified several proteins that differentially responded to the two lipid manipulations and play important roles in visual signaling. Two of these proteins are involved in visual pigment synthesis and show an abundance increase upon lipid unsaturation (Table S5): the chaperone NinaA is required for the synthesis of the visual pigments Rh1 and Rh2,^34^ and the oxidoreductase NinaG is essential for the synthesis of the vitamin A-derived chromophore^35,36^ (Table S5). Conversely, the protein levels of the light-sensitive cation channel Trpl and the eye-specific protein kinase InaC^23^ both decreased upon lipid unsaturation.

In addition to the global proteome analysis, we also quantified the changes in absolute (femtomoles per eye) protein abundances with our targeted MSW method^14^ (Figures 2G and 3H) (Table S3). In the eyes of M2-flies, the molar abundance of phototransduction proteins was significantly lower compared to that of M1-flies, which was very similar to (or not significantly different from) M3-flies. Conversely, the abundance of structural proteins (e.g., actins and Veli) increased within a range of 1.4- to 2.9- fold, consistent with the increased abundance of other proteins of the actomyosin machinery.

Taken together, we analyzed the effects of lipidome manipulations on the ocular proteome in four (M0^8^ and M1, M2, M3) compositionally related semisynthetic diets. We observed that increased unsaturation of the eye lipidome elicited a specific proteome response that differentially changed the abundances of proteins involved in lipid metabolism and transport, muscle organization, stress responses, and visual signaling.

## 4 Discussion

### 4.1 Accounting the Consequences of Dietary Manipulations

Dietary manipulations (e.g., lipid-rich vs low-lipid diet) combined with quantitative *omics* analyses provide insights into complex metabolic responses at the full-organism level. However, in this work, we used dietary interferences to manipulate the lipid composition of an organ, the eye, and to study the consequences for its proteome. Conceivably, pronounced lipidomic alterations could massively interfere with the composition of the membrane proteome, as has been shown in cell culture experiments. To clarify the proteome response, we subtracted proteomic trends that are not unequivocally associated with membrane lipid unsaturation. For example, the major Rhodopsin Rh1 did not match these specificity requirements because its abundance was also reduced by lipidome-independent manipulations (i.e., by the M0 diet). Unexpectedly, we found that the ocular proteome, including its membrane complement, showed only a limited response to membrane unsaturation. Moreover, it was also surprising (although in line with our independent observations of organ lipidomes) that the lipid class composition was apparently unaffected by the unsaturation of membrane lipids.

### 4.2 Muscle-Related Proteins Respond to Lipid Unsaturation in the Eye

We studied the effects of lipidome manipulations on the ocular proteome and emphasized that grouping differentially expressed proteins by their response to lipid unsaturation does not necessarily imply a similar function or molecular relationship. Yet, we identified a group of seven proteins that have muscle-related functions and increase their abundances with ocular lipidome unsaturation (but are unaffected by lipidome saturation). This result can be interpreted in several ways: the differential expression of these muscle-related proteins could reflect a reorganization of muscle tissue in the eye in response to an increased level of lipid unsaturation. For instance, this could involve the ocular muscles that move the *Drosophila* retina to track motion and stabilize the retinal image.^37^ Furthermore, the actomyosin machinery is required for the formation of the luminal matrix space between the rhabdomeres^38^ and actomyosin contraction plays a critical role in shaping the morphology of the eye.^39^ Alternatively, it is conceivable that these proteins have other, yet to be identified, functions in the *Drosophila* eye that are required upon increased lipid unsaturation. Interestingly, the expression of genes that are associated with muscle development has been detected in the pupal eye,^40^ and Troponin I/Wings up A additionally controls the proliferation of epithelial cells as well as the localization of apical-basal polarity signaling proteins.^41^ Third, the upregulation of muscle-related proteins could be due to a yet to be identified mechanistic link between lipid unsaturation and increased muscle protein expression that co-occur at low temperatures; we previously showed that at temperatures below 15 °C, *Drosophila* alter their dietary preference from yeast to plant material in laboratory foods but also in the wild,^5^ which provides unsaturated fatty acids that improve membrane fluidity and motor functions.^1^ Notably, another study revealed that the expression of genes that encode the myosin heavy and light chains is upregulated at low temperatures in adult *Drosophila*, potentially as an adaptive response to compensate for decreased muscle contractility and to maintain flight performance at low temperatures.^42^ These data suggest that the muscle machinery is plastic and can adapt to both dietary and temperature stresses. Since honeybees show cast- and thus task-specific lipid unsaturation differences in their flight muscle membranes,^43^ it is possible that muscles in the fly body are also affected by our lipid manipulations and that the regulation of the unsaturation of membrane lipids is part of a multipurpose and evolutionarily conserved mechanism among insects.

### 4.3 Stress-Responsive Proteins are Downregulated upon Lipid Unsaturation

We also identified two differentially lipid-responsive proteins involved in stress responses, TotA and Smg5, whose abundances decrease with lipid unsaturation. Smg5 is an essential nonsense-mediated mRNA decay factor that was found in an obesity screen as a regulator of TAG levels in muscle cells.^29,30^ Notably, humoral factor TotA showed the strongest (over 4-fold) downregulation among all proteins in response to lipidome unsaturation. In addition, *TotA* expression is induced by various environmental stresses such as UV light, heat, cold,^44^ bacterial infection, and oxidative agents.^28^ Moreover, *TotA* expression is also increased when flies adapt to a high-protein-low-carbohydrate diet.^45^ It is unclear why a broad range of stress stimuli increases *TotA* transcription while lipid unsaturation decreases TotA protein levels. Yet, in another study, a high-fat diet caused *TotA* downregulation specifically in males.^46^ Since TotA is regulated by JAK-STAT and MAPK pathways,^47^ its downregulation could reflect a lower activity of those pathways or an overall reduction in stress load.^48^

### 4.4 Robustness of Ocular Lipid Class Composition and Membrane Protein Expression

*Drosophila* lacks the ability to synthesize long-chain PUFAs from shorter-chain precursors (such as linoleic acid, C18:2), and the rhabdomere membranes lack lipids with PUFA moieties of more than 18 carbon atoms.^4,19^ The TAG that we added in the case of M2 food supplied the omega-3 PUFA DHA, which has not been detected in *Drosophila*(4,49) but is present at high levels in the rhabdomere-equivalent outer segments of mammalian photoreceptors.^50−52^ Unexpectedly, we discovered that once the supplied DHA was metabolized to various shorter-chain polyunsaturated GPLs, the lipid class composition of the eye remained unchanged. Moreover, DHA did not affect the molecular profiles of major sphingolipids (Cer and Cer-PE). Others^53^ and we^1,3,5^ previously observed similar homeostatic trends when flies were switched from a “mostly saturated” yeast diet to a “mostly unsaturated” plant diet. Therefore, we speculate that this robustness of the ocular lipid class composition to dietary changes may be a general homeostatic feature of the organization of eukaryotic tissues, while the length and unsaturation of fatty acid moieties are more variable, potentially allowing a compensatory response toward environmental and dietary challenges.^1,2^ Another unexpected observation was that the increased unsaturation of membrane lipids in the fly eye affected the abundance of only a very few membrane proteins. This robustness of the expression of membrane proteins also suggests that once formed, lipid–protein assemblies can be incorporated into membranes of variable composition and properties.

### 4.5 Effects of Dietary Manipulations on Photoreceptor Morphology and Phototransduction Protein Expression

Photoreceptors have highly specialized light-sensing compartments that are called rhabdomeres in flies and outer segments in mammals.^20^ Altering the molecular composition of the rhabdomere membrane can affect its physical properties, such as stiffness or fluidity^54,55^ and thereby visual signaling:^6^ a PUFA-deficient yeast diet impairs the speed and sensitivity of the phototransduction cascade, potentially due to decreased rhabdomere membrane fluidity.^6^ In a previous study, we discovered that the expression of the components of the rhabdomeric phototransduction machinery is dependent on vitamin A;^8^ in the current study, we found that the machinery is also dependent on lipid unsaturation. The impacts of M2-diet resemble vitamin A deficiency (M0-diet) because both cause a severely reduced rhabdomere size and decreased levels of phototransduction proteins.^8,9^ The M3-diet, which contains three short-chain saturated TAGs, also decreased the phototransduction protein levels but did not cause any obvious rhabdomere damage. This suggests that phototransduction proteins require a specific degree of membrane lipid saturation and that rhabdomere defects are not the cause for the decreased phototransduction protein expression, which is also consistent with our previous finding that *crumbs* mutants have severe rhabdomere defects but do not exhibit significant changes in the levels of phototransduction proteins or Rh1.^22^ The molecular mechanisms that underlie the impacts of these three different dietary manipulations on the expression levels of phototransduction proteins remain to be elucidated.

In conclusion, we anticipate that these insights into the molecular responses of the *Drosophila* eye proteome to specific lipid manipulations and the data sets that we generated will be useful resources for the genetic dissection of the mechanisms that maintain visual function when the eye is exposed to dietary challenges.

## Supporting Information Available

Lipid composition of M1- and M3-foods determined by shotgun mass spectrometry; schematic of phototransduction-related proteins in *D. melanogaster* photoreceptors; and GO term analysis of proteins that responded to lipidome unsaturation (PDF)
Quantification of the ocular lipidome of flies raised on different diets (XLSX)
Quantification of the ocular proteome of flies raised on M1 and M3 diets (XLSX)
Absolute quantification of phototransduction and other target proteins (XLSX)
List of proteins that specifically respond to lipidome unsaturation (XLSX)
Quantification of the ocular proteome of flies raised on M3 and M2 diets (XLSX)
Quantification of the ocular proteome of flies raised on M1 and M0 diets (XLSX)
