# Supplementary material for: Lipidome Unsaturation Affects the Morphology and Proteome of the Drosophila Eye
Source: J Proteome Res. 2024 Mar 14;23(4):1188–99. doi: 10.1021/acs.jproteome.3c00570 (PMC11002927; doi:10.1021/acs.jproteome.3c00570)
Supplement: Supplementary file 1 — pr3c00570_si_001.pdf [file pr3c00570_si_001.pdf]

SUPPORTING INFORMATION

Lipidome unsaturation affects the morphology and proteome of the *Drosophila* eye.

Mukesh Kumar[1,4], Canan Has[1,5], Khanh Lam-Kamath[2], Sophie Ayciriex[1], Deepshe Dewett[2], Mhamed Bashir[2], Clara Poupault[2], Kai Schuhmann[1], Henrik Thomas[1], Oskar Knittelfelder[1], Bharath Kumar Raghuraman[1], Robert Ahrends[3], Jens Rister[2]* and Andrej Shevchenko[1]*

[1] Max Planck Institute of Molecular Cell Biology and Genetics, Pfotenhauerstrasse 108, 01307 Dresden, Germany

[2] Department of Biology, University of Massachusetts Boston, Integrated Sciences Complex, 100 Morrissey Boulevard, Boston, MA 02125, USA

[3] Department of Analytical Chemistry, University of Vienna, 1090 Vienna, Austria

[4] Current address: Cell Signaling Technology, 3 Trask Lane, Danvers, MA 01923, USA

[5] Current address: Centogene GmbH, 18055 Rostock, Germany

* Corresponding authors

jens.rister@umb.edu

shevchenko@mpi-cbg.de

Table of contents

List of Supplementary Figures:

Supplementary Figure S1: Lipid composition of M1- and M3-foods determined by shotgun mass spectrometry.

Supplementary Figure S2: Schematic of phototransduction-related proteins in *Drosophila melanogaster* photoreceptors.

Supplementary Figure S3: GO term analysis of p*roteins* responded to lipidome unsaturation.

List of Supplementary Tables:

Supplementary Table S1: Quantification of the ocular lipidome of flies raised on different diets.

Supplementary Table S2: Quantification of the ocular proteome of flies raised on M1 and M3 diets.

Supplementary Table S3: Absolute quantification of phototransduction and other target proteins.

Supplementary Table S4: List of proteins that respond to lipidome unsaturation.

Supplementary Table S5: Quantification of the ocular proteome of flies raised on M3 and M2 diets.

Supplementary Table S6: Quantification of the ocular proteome of flies raised on M1 and M0 diets.

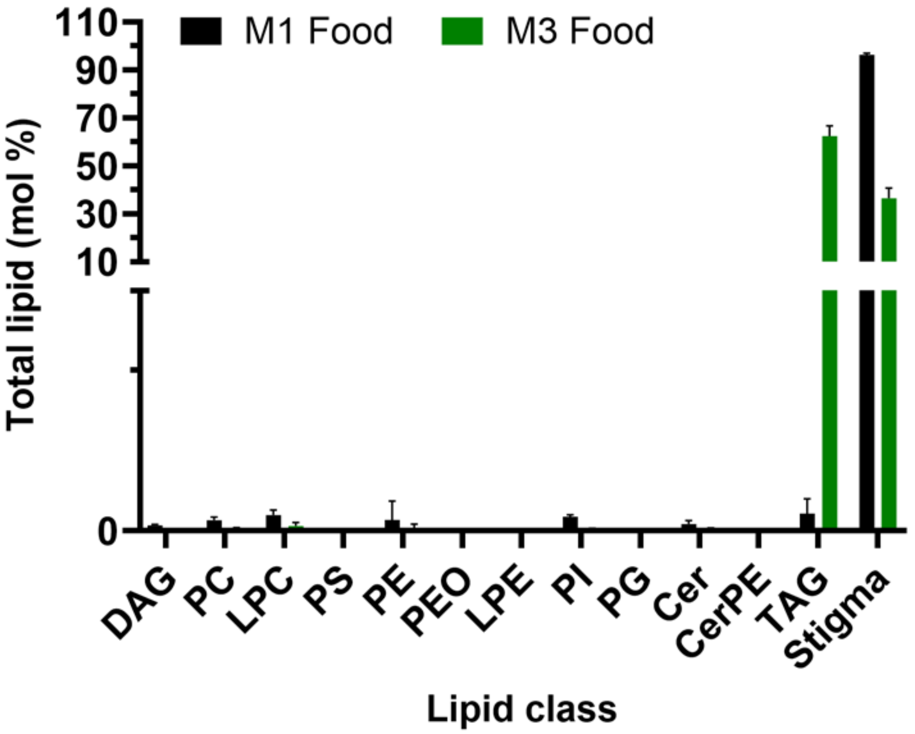

Supplementary Figure S1: Lipid composition of M1- and M3-foods determined by shotgun mass spectrometry. The abundance of lipid classes is shown on the y-axis as mol%. M1-food was prepared on the basis of water-soluble yeast extract supplemented with the phytosterol Stigmasterol. M3-food was prepared by adding a mixture of synthetic saturated TAGs (TAG 42:0; TAG 48:0; TAG 54:0) to M1-food. Note that the amount of Stigmasterol (in mg per gram) is the same in M1 and M3 foods, although in M3-food its mol% content is lower because of the added TAGs. Other lipid classes are residual lipids from the soluble yeast extract. Shotgun lipidomics could not quantify the content of free fatty acids. The mol% composition of M2-food containing unsaturated TAG 66:18 is similar to M1-food and is not shown here.

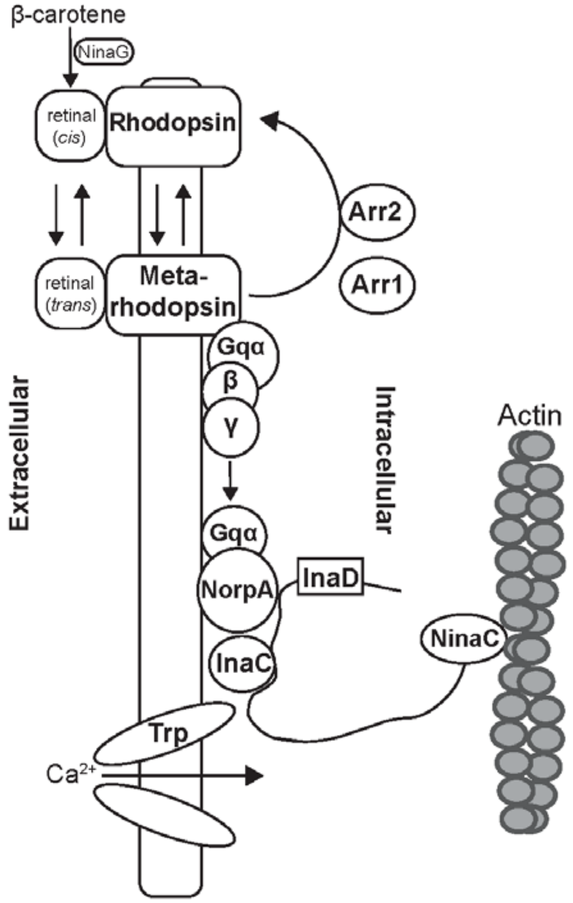

Supplementary Figure S2: Schematic of phototransduction-related proteins in *Drosophila melanogaster* photoreceptors. β-carotene is converted to 11-*cis*-3-hydroxyretinal that covalently binds to a Rhodopsin protein (*e.g.*, Rh1). Absorption of light isomerizes the 11-*cis* to the all-*trans* configuration and converts Rhodopsin to Metarhodopsin. This results in the activation of the Gq protein and the phospholipase C NorpA as well as the opening of Trp channels; the influx of Ca^{2+} depolarizes the photoreceptor neuron. The scaffolding protein InaD forms signaling complexes that include NorpA, Trp, the protein kinase InaC, and the unconventional myosin NinaC. Lastly, InaC (inactivates NorpA) and the Arrestins Arr1 and Arr2 (inactivates Metarhodopsin) terminate the light response.

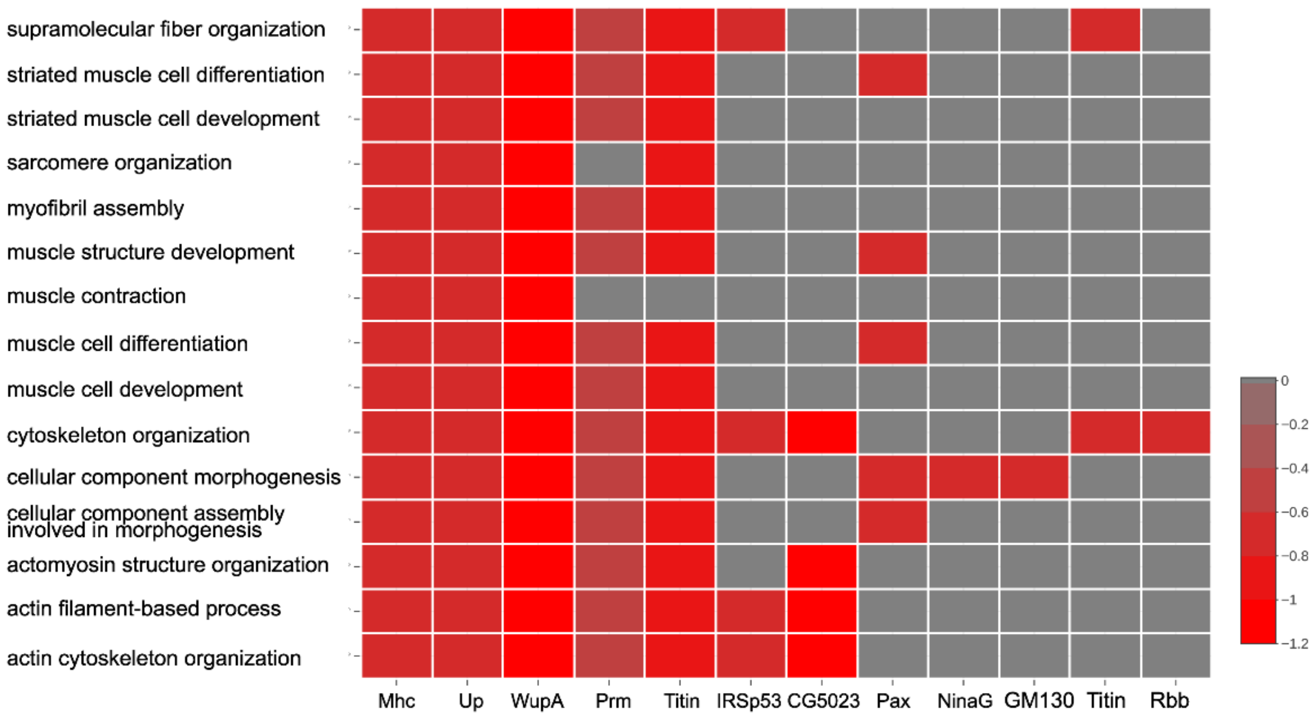

Supplementary Figure S3: **Gene ontology annotation of proteins specifically responding to membrane lipid unsaturation**. Genes were mapped to the *D. melanogaster* gene ontology (biological process) database using the g:Profiler software (v. 0.2.2; database from 29/03/2023). The ontology terms with statistical significance (adj. *p*-value < 0.05) were plotted according to the fold change of the abundance of eye proteins in the M3- vs M2- flies comparison (log2FC M3_M2 in Supplementary Dataset 4S) as interactive heatmap using the R plotly library (v. 4.10.1) (Sievert, 2020) of R (version 4.3).
